# Results of an abbreviated phase-II study with the Akt Inhibitor MK-2206 in Patients with Advanced Biliary Cancer

**DOI:** 10.1038/srep12122

**Published:** 2015-07-10

**Authors:** Daniel H. Ahn, Junan Li, Lai Wei, Austin Doyle, John L. Marshall, Larry J. Schaaf, Mitch A. Phelps, Miguel A. Villalona-Calero, Tanios Bekaii-Saab

**Affiliations:** 1 Divison of Medical Oncology, Ohio State University Comprehensive Cancer Center, 300 W 10th Ave, Columbus, OH 43210; 2 College of Pharmacy, Ohio State University, 500 W 12th Ave, Columbus, OH 43210; 3 Center of Biostatistics, Ohio State University, 300 W 10th Ave, Columbus, OH 43210; 4 Cancer Therapy Evaluation Program, National Cancer Institute, 9609 Medical Center Dr, Bethesda, MD 20892; 5 Division of Hematology/Oncology, Lombardi Comprehensive Cancer Center, 3800 Reservoir Rd, Washington, DC 20057.

## Abstract

Biliary cancers (BC) are rare, chemoresistant and are associated with a poor prognosis. Targeting the Akt pathway is of significance in BC. We hypothesized that the allosteric inhibitor MK-2206 will be active in BC. This was a multi-institutional phase II study of MK-2206 given to patients with advanced, refractory BC. The primary end point was overall response rate. We also characterized pharmacokinetic profiles of MK-2206 in these patients and explored its potential correlation with clinical outcomes. Eight patients were enrolled prior to early termination of the trial. All patients had received prior systemic therapy. The best response observed was stable disease, exceeding 12 weeks in two patients. Toxicities were mild and tolerable. MK-2206 exhibited a pharmacokinetic profile with an apparent slow absorption followed by biphasic elimination in these patients with BC. No significant association was observed between the pharmacokinetic properties of MK-2206 and clinical outcomes. MK-2206 as a single-agent in BC is tolerable with pharmacokinetic properties similar to patients with other solid tumors. No clinical activity was observed in this limited population. Further development of Akt inhibitors may need to focus on combinations with other molecular targeted agents, conventional cytotoxic chemotherapy and prospective patient selection.

Biliary cancers (BC) are rare, chemoresistant and are associated with a poor prognosis. The tumor arises from the ductal epithelium of the biliary tree within the liver (intrahepatic), extrahepatic ducts (extrahepatic) or gallbladder^1^. The mechanisms of cholangiocarcinogenesis are complex and involve multiple molecular signaling pathways and inflammatory cytokines that contribute to tumor growth, chemoresistance and cachexia in biliary cancer^2^,^3^. The current standard regimen for untreated advanced biliary cancer is the combination of cytotoxic chemotherapy with gemcitabine and cisplatin, but the disease is nearly always fatal, with a median survival that remains less than one year^4^. In addition, trials for second-line therapy in refractory biliary cancer have been disappointing, highlighting the urgent need to develop new and effective therapies^5^,^6^,^7^.

The PI3k/Akt pathway is downstream of the common growth factor receptor tyrosine kinases (RTKs), including EGFR, HER2, and IGFR, and is a likely driver of tumor progression in most carcinomas^8^,^9^,^10^. Akt, also known as protein kinase B, is activated in a substantial proportion of human solid tumors (breast, endometrial, ovarian, prostate, pancreatic, gastric and non-small cell lung cancer). Upregulation of Akt can be caused by direct amplification and mutation of Akt or by overexpression of TKR, PI3K and RAS, and/or by inactivation of the tumor suppressor, PTEN^11^,^12^,^13^. Because of its key function in cell survival, Akt plays a pivotal role in rendering tumor cells insensitive or resistant to chemotherapy or targeted agents, making it an increasing area of interest in development of targeted therapies.

Pre-clinical data has shown activated Akt overexpression in biliary cancers and has demonstrated growth inhibition with Akt dephosphorylation^11^,^14^. Treatment of cholangiocarcinoma cell lines with PI3K inhibitor (LY294002) or the MEK 1/2 (UO126) attenuated the effect of CXCL12-induced cholangiocarcinoma cell invasion. These findings indicate that signaling pathways (MEK 1/2 and Akt) are essential for CXCL12-induced cholangiocarcinoma proliferation and cell invasion, implying a potential role for inhibition of Akt and or MEK signaling cascades in the treatment of biliary cancers.

MK-2206 is an oral selective allosteric inhibitor of Akt that targets all three isoforms of human Akt (Akt-1, Akt-2 and Akt-3) with 50% inhibitory concentration (IC_50_) values of 8, 12 and 65 nM, respectively. In a phase I study of solid tumors, MK-2206 demonstrated evidence of target modulation and anti-proliferative activity as a single agent and in combination with other agents^15^. Previous studies have shown that oxidation and/or glucuronidation are the primary elimination pathways of MK-2206. While oxidation is primarily mediated by CYP3A4, it is unknown which UGT enzyme isoforms are responsible for glucuronidation of MK-2206. Interestingly, MK-2206 is not a significant inhibitor or inducer of major CYP enzymes (IC_50_ > 35 μM for CYP3A4, 2C9, and 2D6 inhibition, and has insignificant effect on CYP3A mRNA and activity at 0.1 to 10 μM)^16^, therefore, it is anticipated that MK-2206 does not perpetuate significant drug-drug interactions at the clinical dose levels. This premise is supported by a recent phase 1 study demonstrating that the combination of MK-2206 with standard chemotherapy agents, such as carboplatin/paclitaxel, docetaxel, or erlotinib, does not significantly influence the pharmacokinetic properties and potency of MK-2206 in solid tumors^16^.

Considering these findings, we hypothesized that MK-2206 would be active in patients with advanced, refractory BC as a single-agent or in combination with other standard cytotoxic agents. We also hypothesized that beneficial clinical effects of MK-2206 would correlate with the presence of activation of the PI3K/Akt pathway. We conducted and report here a phase II study of single-agent MK-2206 in BC to evaluate its efficacy and tolerability at a dose of 200 mg given weekly.

## Patients and Methods

The protocol of this study were reviewed and approved by the Ohio State University, Georgetown University and Case Western University Institutional Review Board. The methods were carried out in accordance with the approved guidelines and regulations. Eligible patients were required to have histologically confirmed biliary tract carcinoma that was surgically unresectable. All patients provided written informed consent before the initiation of the study. All patients were required to have either fresh or paraffin-embedded tissue from tumor blocks less than a year prior to enrolling onto the study. Patients had to have measurable disease per Response Evaluation Criteria in Solid Tumors (RECIST)^17^, one prior therapy for metastatic disease and no prior exposure to Akt inhibitors. Patients with prior cryotherapy, radiofrequency ablation, ethanol injection, transarterial chemoembolization (TACE) or photodynamic therapy were included provided that greater than 6 weeks had elapsed and indicator lesion(s) were outside the area of prior treatment. Patients who received prior radiation therapy with or without the use of fluoropyrimidine as a radiosensitizer in the adjuvant setting were allowed if greater than 12 weeks elapsed since therapy. Additional criteria included age ≥18 years, life expectancy ≥12 weeks, Eastern Cooperative Oncology Group performance status ≤2 and the ability to take and absorb oral medications. Patients were required to have normal organ function, including total bilirubin ≤1.5 times the upper limit of normal and AST/ALT ≤2.5 times the upper limit of normal. Selected exclusion criteria included prior treatment with Akt inhibitors; brain metastases; prior recipient of other investigational agents; diabetes mellitus; history of cardiac disorders including arrhythmias or QTc >450 milliseconds for males and >470 milliseconds for females; pregnant women; and HIV infection.

### Study Design

This was a National Cancer Institute (NCI)/Cancer Therapy Evaluation Program (CTEP)-sponsored phase II, open label, multicenter trial led by The Ohio State University with the participation of Georgetown University and Case Western University (ClinicalTrials.gov NCT01425879, date of registration 08/27/2011). MK-2206 was provided by NCI/CTEP. The primary objective of this study was to determine the overall response rate (complete response and partial response) as defined by RECIST^17^. Tumor tissue samples were required from all patients prior to enrollment. Secondary objectives included overall survival (OS), progression-free survival (PFS), and the presence of any mutations in the pI3k/AKT pathway and the evaluation of toxicities related to MK-2206.

### MK-2206 Administration and Dose Modifications

The starting dose and schedule for MK-2206 was 200 mg given weekly in 28-day cycles without interruption. Treatment was administered on an outpatient basis. Two levels of dose reductions were planned (135 mg weekly and 90 mg weekly) with patients taken off the study for additional dose reductions.

### Assessment of Response and Toxicity

Radiologic assessment was done by computed tomography every 8 weeks and responses were measured according to RECIST 1.1^17^. Toxicities were defined by the NCI-Common Terminology Criteria of Adverse Events version 4.0.

### Correlative Studies

#### Pharmacokinetics

Plasma samples were collected from all patients at Week 1 of Cycle 1 immediately prior to dose administration, and at 30 min, 1, 2, 3, 4, 6, 8, 24, 48, and 96 hours after dose administration. The 24, 48 and 96-hour samples were collected on the mornings of days 2, 3 and 5 within 4 hours of the target time (e.g. 24 ± 4, 48 ± 4, and 96 ± 4 hours). Plasma samples were also collected immediately prior to the oral intake of MK-2206 tablet(s) on days 8, 15 and 29 (i.e. Cycle 2, Day 1) so that trough pre-dose levels of MK-2206 could be determined in the following studies. The plasma levels of MK-2206 were evaluated using quantitative liquid chromatography/tandem mass spectrometry (LC-MS/MS) at Merck & Co. Non-compartmental PK parameters were determined for MK-2206 using Phoenix WinNonlin (v6.3, Pharsight, Mountain View, CA).

#### Pharmacogenetics

Formalin-fixed/paraffin-embedded (FFPE) or fresh frozen tissue samples were obtained from all patients prior to enrollment. A biopsy was only required if there was insufficient material for analysis or if it was greater than 1 year from the time of biopsy to enrollment. Genomic DNA was extracted from frozen or FFPE tissue (20 micron slides) using FFPE DNA Purification Kit (Qiagen) according to the manufacturer’s instructions. For each DNA sample, the following four SNPs were analyzed using qPCR-based or PCR/RFLP-based assays as previously described: CYP3A4 *22 (C>T, rs35599367), CYP3A5*3 (A>G, rs776746), ABCB1 C1236T (rs1128503), ABCB1 G2677T/A (rs2032582), and ABCB1 C3435T (rs1045642)^18^,^19^,^20^,^21^. These pharmacogenetic endpoints were correlated to the pharmacokinetic parameters and response status. FFPE tissue samples for genomic DNA extraction were available for six of the eight patients on study.

Although patients were required to have tissue samples available prior to enrollment on the study, genomic DNA extraction was not feasible for two of the eight patients on study due to insufficient quantities of tissue. Due to the early termination of the trial from a loss in the funding mechanism for the study, additional correlative studies to assess pathway activation and tumor genetics were not conducted.

### Statistical Methods

This study was designed using Simon’s two-stage approach; the true overall response rate was set at 5% and 20% under null and alternative hypotheses, respectively. With type-I and type-II error rates both at 10%, a total of 35 patients were needed. If 1 or more responses were seen from the first 13 patients, the study would proceed to the second stage. If four or more responses were observed in the whole cohort, the agent would be considered promising. Pharmacokinetics and pharmacogenetic correlative components were primarily descriptive and exploratory, with findings from each of these to guide future clinical use of MK-2206 by potentially identifying individual patient characteristics that may influence outcomes from therapy.

## Results

Eight patients were enrolled between September 2012 and December 2013 prior to early termination of the trial. Patients were followed until disease progression or death. The trial was ended by the sponsor prior to the interim analysis due to a loss in funding support from Merck & Co., Inc. The probability that the trial will stop early for futility at the end of stage I (interim analysis) was computed by estimating the conditional probability that there will be no response out of the first 13 patients given that the first 8 patients had no response. If the true overall response rate is 5%, the probability of stopping early for futility at the end of stage 1 is 77.4% when no response was observed in the first eight patients. If the true overall response rate is 20%, the probability of stopping early for futility at the end of stage 1 is 32.8% when no response was observed in the first 8 patients.

Patient characteristics are listed in Table 1. Of the 8 patients enrolled, 8 were evaluable for response and toxicity. Of the patients enrolled into the trial, six (75%) patients had intrahepatic cholangiocarcinoma while 2 (25%) patients had extrahepatic cholangiocarcinoma. Seven patients (88%) had metastatic disease, and all the patients enrolled into the trial were exposed to prior chemotherapy.

### Treatment Toxicity

The most common toxicities (Table 2) included lymphopenia (75%), rash (63%), fatigue (50%), fever (50%), vomiting (50%) and diarrhea (50%). Toxicities were mostly grade 1 or 2 and were reversible. Only one patient suffered a grade 4 toxicity (hyponatremia). This was felt to be unrelated to MK-2206. All toxicities were manageable and reversible. All 3 patients who experienced grade 3 rash required dose reductions.

### Treatment Efficacy

A median of 2 cycles was administered per patient (range, 1 to 2). No patients had an objective response. The best response for all patients accrued in the study was stable disease (SD) lasting more than 12 weeks that was observed in two patients (Table 3). Median PFS was 1.7 months and median OS was 3.5 months.

### Pharmacokinetics

**Figure 1:**
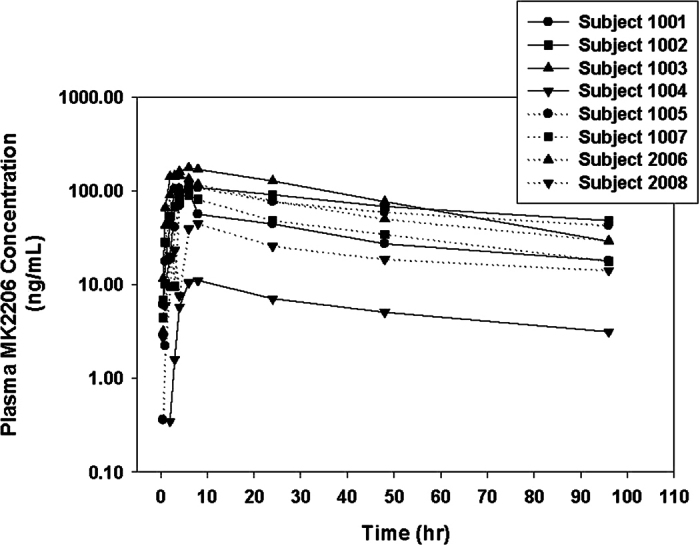
MK-2206 plasma concentration profiles following the oral administration of the first dose for each patient. Pharmacokinetic profile for MK-2206 is for each subject after receiving their first dose of MK-2206.

After oral administration of the first dose of MK-2206 (200 mg weekly), plasma samples from eight patients were collected and the concentrations of MK-2206 were determined. Plasma concentration-time curves of these patients are shown in Fig. 1. The median time to peak observed plasma concentrations (Tmax) was 6 hrs (range 4 to 8 hrs). After reaching maximum observed concentration (Cmax), MK-2206 plasma concentrations declined in a biphasic manner with a median terminal elimination half-life of 58 hrs (range 34 to 88 hrs). Table 4 summarizes the PK parameters of MK-2206 from non-compartmental analyses after the first dose. Of note, the Cmax and AUC for subject 1004 is significantly lower compared to the other seven subjects (1004 has approximately 6% to 25% the Cmax and AUC compared to others), suggesting a significantly higher clearance or lower absorption in this patient relative to others. The mechanisms underlying the unique PK properties of Subject 1004 remain unclear. While several potential explanations exist for the difference, no evidence of sampling errors could be identified, nor were co-medications deemed to be relevant for drug-drug interactions. One notable characteristic of subject 1004 is that this patient is of East Asian decent, while the other 7 patients were Caucasian. Differences in metabolism between East Asians and Caucasians are common, especially in the activity of several phase I enzymes including CYP2D6 and the CYP2C subfamily^22^.

We also measured the trough pre-dose levels of MK-2206 in these patients at days 8 and 15. The average plasma concentrations of MK-2206 in these patients at days 8 and 15 were 14.7 +/− 10.6 ng/mL and 14.4 +/− 6.9 ng/ML, respectively. This data suggests there is no significant accumulation of MK-2206 in patients with the current regimen.

Overall, the PK properties of MK-2206 in these patients (excluding subject 1004) were comparable to those previously reported (Supplementary Table A)^23^. Additionally, the 48-hr plasma concentrations of MK-2206 in 6 of these 8 patients were higher than 22.7 ng/mL (i.e. 56.8 nM), for which this or higher plasma concentrations were reported to be effective in patients with breast cancer^24^. Plasma concentrations measured at 48 hours (hrs) for the other two patients were 18.5 ng/mL and 5.06 ng/mL (subject 1004). The relevance of this threshold plasma concentration could not be evaluated given the small number of patients treated on this trial.

### Pharmacogenetics

While not a significant inhibitor or inducer of major CYP enzyme activity, MK-2206 is converted to oxidative metabolites in human microsomes via CYP3A4 and is a substrate of P-glycoprotein. Hence, we determined genotypes of selected SNPs in CYP3A4, CYP3A5, and ABCB1, including rs35599367 (CYP3A4), rs776746 (CYP3A5), rs1128503, rs2032582, and rs1045642 (ABCB1). The results are listed in Table 5. While all six tested patients were homozygous (GG) for CYP3A5 rs776746, Subject 1005 was heterozygous for CYP3A4 rs35599367 (CT). Moreover, Subjects 1001 and 1003 were heterozygous for all three ABCB1 SNPs (rs1128503, rs2032582, and rs1045642), whereas the genotypes of these three SNPs in Subject 1002 were homozygous (TT). However, there is no apparent association between the PK properties and the genotypes of these five SNPs in this small patient population.

## Discussion

Biliary cancer remains a challenging cancer with a universally poor prognosis. The rationale for Akt inhibition was based on the demonstration of a potential role for Akt/PI3k signaling pathways in the carcinogenesis of biliary cancer. Findings from previous pre-clinical studies have shown Akt and MAPK overexpression in biliary cancer cell lines, suggesting Akt inhibition as a potential role for treatment^25^.

While the study was inconclusive due to its premature termination, our preliminary findings suggest that the oral Akt inhibitor, MK-2206, did not demonstrate meaningful clinical activity as a single-agent in patients with refractory biliary cancer at the doses administered. While no patients had an objective response, two patients experienced prolonged SD (>12 weeks) prior to early closure of the trial and before the interim analysis. Although none of the patients experienced an objective response, the premature termination of the trial did not allow us to fully assess the true efficacy and potential benefit of MK-2206 in biliary cancer. PFS could be a more desirable endpoint for studies incorporating single agent biologics. However, in the absence of randomization, measuring objective response rate may be more meaningful, especially given recent data with other targeted agents in advanced biliary cancers demonstrating objective responses in this disease population^26^,^27^. Taking into account the statistical design of the study, the probability of observing at least one response in the next five patients in the first stage of the study, which would allow the study to proceed to the second-stage was 22.6% to 67.2% when the true response rate ranges between 5% and 20%.

The plasma pharmacokinetics of weekly MK-2206 at a 200 mg dose in patients with biliary cancer is similar to that reported in patients with other solid tumors, including breast cancer and malignant glioma^15^,^23^,^24^. For example, the average Cmax value achieved after the first dose in biliary cancer patients (excluding subject 1004) was 114 (+/− 41.2 ng/ mL), whereas the Cmax in a group of children with refractory malignancies was 171 (+/− 89.3 ng/mL). Moreover, there is no substantial difference in BSA-adjusted CL/F between biliary cancer patients (16.5 +/− 7.2 L/hr/m^2^) and children with refractory malignancies (18.0 +/− 18.8 L/hr/m^2^). The limited pharmacogenetic data available for CYP3A4, CYP3A5, and ABCB1 did not reveal any major factors associated with drug disposition. In summary, the plasma exposures in these biliary cancer patients were similar to those reported in other disease populations where more clinical activity was observed. Therefore, the lack of observed efficacy achieved in these 8 patients was not due to differences in whole-body disposition of MK-2206. A high incidence of grade 3 rash (38%) was observed in patients who received MK-2206. Despite plasma pharmacokinetics being similar in these patients to previous reports, it is unclear if plasma drug levels are representative of tumor site concentrations. Drug exposure at the tumor site could be significantly lower due to poor access to biliary tumors or by metabolic/catabolic influences acting locally within the hepato-biliary region surrounding the tumor. Further understanding of this will be important for further development of MK-2206 combination therapy in this disease population.

The absence of response may be related to the lack of an improved selection of patients. There is a noticeable absence of potential biomarkers for MK-2206 that could have helped with this process. It is possible that a select subset of patients with phosphorylated Akt (pAkt) overexpression may benefit from MK-2206 and previous studies demonstrated a substantial proportion of patients with advanced biliary cancer exhibit pAkt overexpression^26^. The clinical efficacy of Akt inhibition in advanced biliary cancers with pAkt overexpression may merit furhter investigation in future studies. Additionally, the lack of response to MK-2206 may be due to co-activation of Akt and MAPK signaling pathways within the tumors. Previous studies indicated that the co-activation and cross talk between the Akt/PI3k and MAPK pathways constitute a potential mechanism of resistance to Akt monotherapy^28^,^29^,^30^. High levels of MEK can lead to resistance to Akt inhibition, thus, even with adequate Akt inhibition, cells with MEK or ERK overexpression may continue to influence several of these downstream proteins and thus prevent apoptosis and promote cellular proliferation^26^. In Akt resistant cells, MEK inhibition restores susceptibility to Akt inhibition, suggesting negative modulation of the MAPK pathway may be a key to overcoming resistance to Akt inhibitors^29^. With previous studies showing promising single-agent activity with MEK inhibition in biliary cancer^26^, future studies combining Akt and MEK inhibitors may be warranted in patients with biliary cancers. Lastly, Akt overexpression has been associated with resistance of biliary cancer cells to chemotherapy and radiotherapy, suggesting a potential role for Akt inhibitors in combination with cytotoxic treatments^16^. Furthermore, *in vitro* studies have demonstrated a decreased sensitivity to allosteric Akt inhibitors in cell lines with somatic mutations in Akt, suggesting that appropriate screening and selection of patients without Akt mutations may have an improved clinical benefit from MK-2206^31^.

In conclusion, MK-2206 as a single-agent in biliary cancer was generally tolerable, although no objective clinical activity was observed in this small sample of patients. This is the first report of the use of Akt-targeted therapies in biliary cancers. Monotherapy targeting Akt inhibition may have clinical relevance for a subset of patients with biliary cancer, however further studies assessing the utility of MK-2206 in combination with other molecular targeted agents or in conjunction with conventional cytotoxic chemotherapy are warranted. Correlative investigation confirming the potential benefit in responders and identifying new positive predictors of clinical response are needed to better understand the mechanisms of activity and to select patients that will derive benefit from treatment.

## Additional Information

**How to cite this article**: Ahn, D. H. *et al.* Results of an abbreviated phase-II study with the Akt Inhibitor MK-2206 in Patients with Advanced Biliary Cancer. *Sci. Rep.* **5**, 12122; doi: 10.1038/srep12122 (2015).

## Supplementary Material

Supplementary Information

## Figures and Tables

**Table 1:** Patient Demographics and Characteristics.

**Characteristics**	**No. of patients (n = 8)**
**Sex**	
Male	4
Female	4
**Age**	
Median	58
Range	40–83
**Race/ethnicity**	
White	7
Asian	1
**ECOG Performance Status**	
0	2
1	6
**Prior treatment**	
Surgery	0
Chemotherapy	8
Metastatic	8
Adjuvant	0
Gemcitabine-based	8
Other	0
Biologic therapy	0
**Disease Site**	
Intrahepatic	6
Gallbladder	0
Extrahepatic	2
**Stage**	
Locally advanced	1
Metastatic	7

**Table 2:** Common Toxicities (n = 8).

**Toxicities**	**All toxicities (%)**	**Grade 1 (%)**	**Grade 2 (%)**	**Grade 3 (%)**	**Grade 4 (%)**
Anemia	12.5	12.5			
Constipation	12.5	12.5			
Thrombocytopenia	25	12.5		12.5	
Diarrhea	50	25	12.5	12.5	
Dry Mouth	25	25			
Dry Skin	38	25	12.5		
Fatigue	50	25	25		
Fever	50	25			
Hand Foot Syndrome	38	25	12.5		
Hypophosphatemia	25		25		
Leukopenia	12.5	12.5			
Lymphopenia	75	25	12.5	37.5	
Macular Rash	63	12.5	12.5	38	
Mucositis	38	38			
Nausea	38	38			
Vomiting	50	50			
Hyponatremia	12.5				12.5

**Table 3:** Efficacy Results.

**Subject**	**Primary site**	**Best response**	**Progression-Free Survival**	**Overall Survival**
1001	Intrahepatic	Progressive disease	1.8	2.2
1002	Intrahepatic	Progressive disease	0.5	3.1
1003	Intrahepatic	Progressive disease	1.2	3.8
1004	Intrahepatic	Progressive disease	1.6	3.1
1005	Intrahepatic	Stable disease	3.4	20.2
1007	Extrahepatic	Progressive disease	6.6	6.7
2006	Intrahepatic	Progressive disease	1.2	2.8
2008	Extrahepatic	Stable disease	5.6	6.1

**Table 4:** Summary of MK-2206 Pharmacokinetic Parameters from Non-compartmental Analyses.

**Subject**	**λz (1/hr)**	**Half-life (hr)**	**Tmax (hr)**	**Cmax (ng/mL)**	AUC_0–96_ (hr*ng/mL)	**Vz/F (L)**	**CL/F (L/hr)**
1001	0.013	53.3	4	106	3322	3270	42.5
1002	0.009	80.4	6	111	6850	1867	16.1
1003	0.021	33.8	6	176	8446	989	20.3
1004	0.011	62.5	8	11.1	531	22150	245
1005	0.008	86.9	8	108	6018	2208	17.6
1007	0.014	50.2	6	90.1	3734	2883	39.8
2006	0.013	52.4	4	144	5854	1874	24.8
2008	0.008	87.5	8	44.7	2061	6564	52.0

**Table 5:** Genotypes of patients*.

**Subject**	**CYP3A4** *22 (C→T) (***rs35599367***)	**CYP3A5** *3 (A→G) (***rs776746***)	**ABCB1 Exon12 C1236T (*****rs1128503***)	**ABCB1 Exon21 G2677T/A (*****rs2032582***)	**ABCB1 Exon26 C3435T (*****rs1045642***)
1001	CC	GG	CT	GT	CT
1002	CC	GG	TT	TT	TT
1003	CC	GG	CT	GT	CT
1005	CT	GG	CC	GG	CC
1007	CC	GG	TT	GG	CT
2008	CC	GG	CC	GT	CT

*DNA from Subjects 1004 and 2006 were not available due to limitations in sample quantity.
