# Supplementary material for: Results of an abbreviated phase-II study with the Akt Inhibitor MK-2206 in Patients with Advanced Biliary Cancer
Source: Sci Rep. 2015 Jul 10;5:12122. doi: 10.1038/srep12122 (PMC4894406; doi:10.1038/srep12122)
Supplement: Supplementary Information [file srep12122-s1.doc]

**Results of an abbreviated phase-II study with the Akt Inhibitor MK-2206 in Patients with Advanced Biliary Cancer**

Daniel H. Ahn^1^, Junan Li^2^, Lai Wei^3^, Austin Doyle^4^, John L. Marshall^5^, Larry J. Schaaf^2^, Mitch A. Phelps^2^, Miguel A. Villalona-Calero^1^, Tanios Bekaii-Saab^1^

**Supplementary Table A**. Comparison of MK-2206 pharmacokinetic parameters

|  | Number of subjects* | Dose level**  (mg/m^2^) | Half life (hr) | Tmax  (hr) | Cmax  (ng/mL) | Vz/F/m^2^  (L/m^2^) | CL/F/m^2^  (L/hr/m^2^) |
| --- | --- | --- | --- | --- | --- | --- | --- |
| OSU Trial | 7 | 110±9.0 | 63.5±21.2 | 6.0±1.6 | 114±41.2 | 1521±931 | 16.5±7.2 |
| Fouladi et al. | 9 | 120 | 44.2±23.3 | 5.1±1.6 | 171±89.3 | 1100±1020 | 18.0±18.8 |
|  |  |  | *p* = 0.11 | *p* = 0.28 | *p* = 0.14 | *p* = 0.41 | *p* = 0.85 |

*Data from Subject 1004 was excluded.

**The dose was normalized to body surface areas for comparison between studies. In both trials, MK-2206 was orally administrated weekly. Pharmacokinetics of MK-2206 were assessed for the first dose in both studies. In the current trial, the initial dose for each patient was 200 mg, whereas patients in the study reported by Fouladu and colleagues received their first doses adjusted for BSA.
